# Recent Advances in Placenta–Heart Interactions

**DOI:** 10.3389/fphys.2018.00735

**Published:** 2018-06-14

**Authors:** Cheryl L. Maslen

**Affiliations:** Knight Cardiovascular Institute, Department of Molecular and Medical Genetics, Oregon Health & Science University, Portland, OR, United States

**Keywords:** congenital heart defects, genetic risk factors, placental insufficiency, heart–placental axis, fetal environment

## Abstract

Congenital heart defects (CHD) occur in ∼1 in every 100 live births. In addition, an estimated 10% of fetal loss is due to severe forms of CHD. This makes heart defects the most frequently occurring birth defect and single cause of in utero fatality in humans. There is considerable evidence that CHD is heritable, indicating a strong contribution from genetic risk factors. There are also known external environmental exposures that are significantly associated with risk for CHD. Hence, the majority of CHD cases have long been considered to be multifactorial, or generally caused by the confluence of several risk factors potentially from genetic, epigenetic, and environmental sources. Consequently, a specific cause can be very difficult to ascertain, although patterns of associations are very important to prevention. While highly protective of the fetus, the *in utero* environment is not immune to insult. As the conduit between the mother and fetus, the placenta plays an essential role in maintaining fetal health. Since it is not a fully-formed organ at the onset of pregnancy, the development of the placenta must keep pace with the growth of the fetus in order to fulfill its critical role during pregnancy. In fact, the placenta and the fetal heart actually develop in parallel, a phenomenon known as the placenta–heart axis. This leaves the developing heart particularly vulnerable to early placental insufficiency. Both organs share several developmental pathways, so they also share a common vulnerability to genetic defects. In this article we explore the coordinated development of the placenta and fetal heart and the implications for placental involvement in the etiology and pathogenesis of CHD.

## Introduction

Congenital heart defects (CHD) are the most common form of birth defect in humans (van der Bom et al., 2011). Despite the recognition that there is a strong genetic component, the etiology is complex and is generally not well understood. Model organisms have taught us a great deal about cardiovascular development and the contribution of specific genes to the creation of the mammalian four chambered heart (Andersen et al., 2014). However, in humans, heart defects are seldom caused by single gene abnormalities. Even in the case of families with a strong inheritance pattern of CHD primarily caused by a single gene defect there is often variable expression or incomplete penetrance. Regardless, familial CHD is the rare exception. The majority of CHD occurs as a sporadic, non-syndromic event, known to geneticists as a simplex trait. The cause is difficult to determine, although there is now evidence that deleterious *de novo* mutations and copy number variants (segments of duplication or deletion of chromosomes) account for up to 15% of CHD, albeit with extreme gene locus heterogeneity and a lack of distinct genotype-phenotype correlations (Yuan et al., 2013). Environmental factors are also known to cause CHD. It is often noted that the heart is the first organ to develop in the human embryo, which makes it susceptible to early environmental insults that may occur before the pregnancy is realized. Epidemiological studies have pointed to many external environmental exposures that lead to CHD as well as other birth defects (Krauss and Hong, 2016). Indeed, environmental factors such as true teratogens have long been suspect in the etiology of CHD. Well known exposures, such as maternal alcohol consumption, which causes fetal alcohol spectrum disorder, may account for thousands of cases of CHD each year (Burd et al., 2007). However, teratogenic exposure likely causes only a fraction of CHD in western countries. Another type of environmental exposure, specifically defects primary to the fetal environment, is an equally important yet less studied factor that contributes to the etiology of CHD. Generally thought to be a highly protected environment, the gatekeeper, which is the placenta, plays a critical role in providing the fetus with all of its basic needs. Here we explore the role of the placenta in the development of the heart and the potential for placental insufficiency to be an important factor in the control of proper cardiovascular development and resulting CHD.

## The Placenta–Heart Genetic Axis

The parallel development of the placenta and the heart has received less attention than the genetics of heart development, yet it may have particular implications for the genetic etiology of CHD. The heart and placenta share key developmental pathways, which suggests that deleterious defects in any of these genes would likely result in perturbations of both heart and placental morphogenesis (Hemberger and Cross, 2001). This could have a synergistic effect, with placental insufficiency exacerbating the inherent developmental defect in the heart. The canonical Wnt/β-catenin pathway is one of the crucial pathways shared simultaneously by the developing heart and placenta. In the developing heart, Wnt signaling is required for myocardial specification, cardiac morphogenesis, valve formation, and proliferation of endothelial and vascular smooth muscle cells (Gessert and Kuhl, 2010). Experimental mis-expression of the Wnt-mediated genes *Hex* and *Islet-1* in mouse models using exposure to teratogens such as lithium or alcohol results in cardiac and placental anomalies (Linask, 2013). These experiments do not tease out the respective contributions of placental insufficiency and primary cardiogenesis disruption to the final manifestation of CHD, but it does raise the intriguing prospect that the severity of the malformation might be mediated by the placenta. It is known that placental blood flow is an important determinant of early embryonic cardiac function, and disruption of vasculogenesis and vascular function can have a deleterious effect on maternal-fetal circulation.

A second network that is critical to both placenta and heart development is made up of the pathways involved in folate metabolism (Hemberger and Cross, 2001). Folate deficiency has long been associated with the incidence of birth defects, to the point where in 1998 the U.S. Food and Drug Administration mandated that all cereal grain products be fortified with folic acid in an effort to reduce the occurrence of these devastating malformations. This was particularly successful in reducing neural tube defects, which was the specific target. Since then it has been recognized that a genetic variant in the methylenetetrahydrofolate reductase (*MTHFR*) gene reduces the mother’s ability to properly metabolize folic acid by 40–60%, resulting in homocysteinemia thereby increasing the risk of birth defects despite the consumption of otherwise adequate levels of folic acid. Maternal carriers of the *MTHFR* risk allele have an increased risk for developing preeclampsia, demonstrating a direct effect on placental health. The genetic variant is also clearly associated with increased risk to the fetus for developing birth defects, particularly neural tube defects (Yadav et al., 2015). However, the risk of CHD due to folate deficiency is not as well established. Several population-based studies show an increased risk for CHD associated with the *MTHFR* risk allele, mostly in the form of septal defects, while others show no association (Mamasoula et al., 2013). This discrepancy may be due, at least in part, to the genetic diversity of populations studied. Some racial/ethnic groups may have a higher risk profile than others. However, it is known that folate is protective of the placental–heart axis against environmental insults (Linask, 2013). This may put some populations at greater risk for having folate-related CHDs than others which may account for some of the discordant findings in population-based association studies.

In addition to shared developmental pathways between the placenta and the heart there is evidence of a transition of developmental control from embryo to placental of cardiomyocyte proliferation and ventricular wall expansion (Shen et al., 2015). Proper development of ventricular wall is essential to heart function as it provides the force for cardiac ejection. The ventricular wall starts to form early in development, prior to the onset of placenta function. IGF2 has been identified as the mitogen responsible for controlling ventricular cardiomyocyte proliferation in the development of the ventricular wall (Li et al., 2011). However, although there is a single mitogen as the major source of control over ventricular growth, the development occurs in two independent stages in mice. The first stage, which occurs prior to the onset of placental function, is epicardial in origin. The signaling takes place in the context of hypoxia and very low glucose, and uses cytokine erythropoietin (Epo) produced by the liver as the regulator of epicardial *Igf2* expression. This, however, switches with the onset of placental function, which begins to provide oxygen and glucose. This fetal environmental change results in a genetic switch where Epo expression by the liver is reduced as the levels of glucose and oxygen become sufficient to support epicardial *Igf2* expression. This fundamental interplay between the developing heart and placenta is a striking example of the complexity of the heart–placenta axis. Overexposure to glucocorticoids, or stress hormones, is known to be detrimental to fetal growth and development by interfering with placental vascularization (Wyrwoll et al., 2016). The fetus and placenta is generally in a low-glucocorticoid environment thanks to high levels of expression of fetoplacental 11β-hydroxysteroid dehydrogenase-2 (11β-HDS2). Mouse models deficient for the 11β-HDS2 gene, *Hsd11b2*, have now been used to look beyond the placental insufficiency to the effect on cardiovascular development (Wyrwoll et al., 2016). This study once again emphasizes the importance of the placental–heart axis. In addition to the previously described effects on placental development it was noted that there was also impaired cardiac functional maturation, as indicated by a failure of the expected gestational increase in umbilical vein blood velocity in spite of the lack of gross morphological changes in the heart. Interestingly, the effect of excess glucocorticoid exposure in the fetal environment, including placental insufficiency, fetal growth restriction, and reduced cardiac function, was reversed by administration of pravastatin, which increases placental vascular growth factor A. Hence, this study not only reinforces the importance of the placenta–heart axis, but also indicates that it can be manipulated to ameliorate poor fetal outcomes.

## CHD as a Secondary Defect to Placental Insufficiency

Not surprisingly, chronic placental insufficiency has serious consequences for the fetus. It has long been recognized that impaired feto-placental circulation often results in restricted fetal growth (Cetin and Antonazzo, 2009), most often referred to as intrauterine growth restriction (IUGR). IUGR is a leading cause of neonatal deaths, and those that do survive usually face life-long health issues. In population studies IUGR has been shown to be associated with congenital malformations, including CHD (Cohen et al., 2016), although there are confounding factors that bring into question a causative association. In particular, IUGR is frequently found in the context of aneuploidy, where it is unknown if the IUGR predisposed the fetus to CHD, or if IUGR and CHD co-occur due to common etiologic factors (Khoury et al., 1988). However, other studies have provided more direct evidence that placental insufficiency may contribute to the cause of CHD. A recent study comparing placental sufficiency in pregnancies with fetal CHD compared to healthy pregnancies using non-invasive placental perfusion imaging showed that there was significantly decreased global placental perfusion and increased regional variation in perfusion that progressed with advancing gestational age when compared to healthy pregnancies (Zun et al., 2017). This suggests that as the demands by the fetus on the placenta increased, the ability of the placenta to meet those demands was progressively decreasing. In another study, the number of terminal villi, as well as the vascular area and density were reported to be small at the end of pregnancies with hypoplastic left heart, which is one of the most clinically severe CHD in live births (Jones et al., 2015). A study of fetuses with a single umbilical artery (SUA) showed that 13% of those fetuses had a cardiac anomaly, including hypoplastic left heart, coarctation of the aorta, tetralogy of Fallot, hypoplastic right heart, pulmonary atresia/stenosis, absent ductus venosus with cardiomegaly, left isomerism, right isomerism, and transposition of the great arteries (Araujo Junior et al., 2015). These were also isolated heart defects as the study excluded cases with chromosomal abnormalities or extracardiac defects. Surprisingly, only 9.8% of the cases of SUA had IUGR and 8.7% had delivery before 34 weeks. Other similar studies have shown association between SUA and CHD, with varying outcomes depending on the study design (Kasznica et al., 1991; Nakayama et al., 1998; Prefumo et al., 2010; Chen et al., 2016). However, none of these studies have the capability of discerning cause from effect.

Unlike many human studies, animal models have the capacity to shed light on molecular mechanisms underlying heart defects occurring specifically due to placental insufficiency. Germline ablation of placental development genes, where the gene is absent only in the placenta, have clearly demonstrated the essential role of placental function in heart development. Cardiac defects occur when p38α-mitogen activated protein (MAP) kinase or peroxisome proliferation-activated receptors (PPARs) are ablated only in the placenta (Linask, 2013). The resulting abnormal placental development and trophoblast invasion resulted in hypoxia and reduced nutritional support of the fetus, which correlated with the occurrence of CHD. Furthermore, rescue of placental function prevented the development of a heart defect (Barak et al., 1999; Adams et al., 2000). In another study, the developmental role for the homeobox gene, *HOXA13*, was delineated in a mouse model (Shaut et al., 2008). Expression data showed that Hoxa13 is highly expressed in the allantoic bud mesoderm, which is the earliest developing component of the placenta. It was not expressed in the cardiac crescent in the earliest stages of heart development. Even so, *Hoxa13* knockout mice, which displayed compromised labyrinth vessel branching and endothelial specification, had CHD that is secondary to the placental insufficiency. In particular, there was substantial thinning of the ventricular wall in the homozygous mutant mice, confirming that placental function plays a significant role in cardiac development.

Another example shows that extraembryonic expression of the *SUMO-specific protease 2 (SENP2)* is required for normal heart development, and that the cardiac defects that occur with inactivation of SENP2 are secondary to placental insufficiency (Maruyama et al., 2016). In this study, conditional knockout of *SENP2* in the developing placenta results in trophoblast defects that are similar to those seen in a global knockout of *SENP2*. In both cases there is resultant CHD with hypoplastic chambers, thinning myocardium, and an absence of atrioventricular endocardial (AV) cushions. Loss of the AV cushions leads to failure of proper septation of the heart (Miquerol and Kelly, 2013). However, if *SENP2* is deleted in the endothelial cells in the fetus, the AV cushions are normal indicating that the heart defects are secondary to placental insufficiency alone. These and other studies make it indisputable that CHD can be the result of placental defects that compromise fetal development. However, not all forms of placental insufficiency result in the same type of CHD. This is likely due to the timing of the insult to the fetus, but also the nature of the impairment.

## Hypoxia

Hypoxia is considered to be a major stressor in fetal development, and can lead to IUGR, fetal loss, low birth weight, CHD, and persistent pulmonary hypertension in the newborn. Hypoxic fetal stress can result from maternal hypoxia, such as would occur through prolonged exposure to high altitude, a period of severely restricted breathing, or even maternal smoking. Not only does maternal hypoxia directly deprive the fetus of oxygen, but there is evidence that it affects placental function. In the developing placenta, hypoxia can lead to cell death and has been shown to impair development of the vascular bed (Kingdom et al., 2000). Structural changes to the placental vasculature that include a decreased diffusion distance from maternal to fetal blood, or a decrease in available oxygen transfer density will enhance the hypoxic environment of the fetus that persists even if maternal hypoxia is reduced. Not only does hypoxia change placental development, but it also changes gene expression in the developing heart which can have profound effects on cardiac morphogenesis, resulting in CHD. One example is the hypoxia-driven disruption of the vascular morphogen, vascular endothelial growth factor (VEGF). VEGF expression is known to be quickly and robustly increased in response to hypoxia. Modeling of cardiac development in response to hypoxia showed that ischemia-induced premature induction of VEGF expression prevented formation of endocardial cushions, which are the endocardium-derived anlagen of the heart septa and valves (Dor et al., 2001). Since cardiac septal defects are the most common form of CHD in humans this study defines an important molecular link between hypoxia and CHD. In addition, there is evidence that there is an important role for localized low oxygen microenvironments in the developing heart that must interact with normoxic microenvironment developmental partners for normal heart development to occur. It has been shown in the mouse that cells in the heart tube in very early heart development are actually meant to be hypoxic, while the cardiac progenitor cells in the second heart field (SHF) are normoxic. It is thought that spatial differences in oxygenation serve as a signal that controls cardiac development by coordinating cell morphogenesis and migration. Disruption of that carefully orchestrated interaction between developmental microenvironments by making cells in the SHF hypoxic results in CHD (Yuan et al., 2017). Observed defects were diverse and included ventricular septal defects (VSD), double outlet right ventricle (DORV), thinning of the ventricular wall, right ventricle hyperplasia, right ventricle dilation, persistent truncus arteriosus, and complex heart defects such as overriding aorta with VSD. In general, the greater the level of hypoxia the more severe the heart defect.

## Chronic Diseases

### Diabetes

Infants of diabetic mothers (IDM) are at a greatly increased risk for having a CHD. Indeed, pregestational maternal diabetes is considered to be an independent risk factor for clinically severe cardiovascular malformations with increased mortality (Loffredo et al., 2001). Although diabetes-associated CHD prevalence estimates vary depending on the study, there was a consistent ≥fourfold increased risk of CHD for IDM compared to offspring of non-diabetic mothers across studies (Oyen et al., 2016). There was no significant difference between type 1 (insulin-dependent) and type 2 (insulin-independent) diabetes mellitus and risk for having a child with CHD. However, according to recent study of a Danish nationwide cohort of over 2 million pregnancies, risk increases to nearly eightfold for women with previous acute diabetes complications compared to mothers without diabetes (Oyen et al., 2016). Neither maternal age at diabetes onset nor duration of diabetes were associated with increased risk. The associated CHD phenotypes are varied, but there was an increased risk for all CHD subtypes. However, the highest risk was for cardiac outflow defects, with the greatest risk for truncus arteriosus and double outflow right ventricle. Transposition of the great arteries and hypoplastic left heart also occurred. In terms of cardiac septal defects, the risk for atrioventricular septal defects (AVSD) was higher than for atrial or ventricular septal defects (ASD, VSD). Likewise, gestational diabetes has been shown to confer risk of heart defects, with a twofold to threefold increased risk seen for large for gestational age babies (Leirgul et al., 2016). Overall, all forms of diabetes are a significant risk factor for all CHD.

The precise nature of the teratogenic effect of diabetes is not fully understood. However, the association between maternal diabetes and CHD appears to be centered in the placenta (Huynh et al., 2015). Structural and functional changes in the placenta of a diabetic mother are known to occur despite the type of diabetes and the level of hyperglycemic control. The surface area of the placenta is increased, particularly in the periphery of the chorionic villous tree. The trophoblastic basement membrane is thickened, which increases the diffusion distance between the maternal and fetal circulations, reducing the rate of nutrient and waste product transfer. However, there is still an increased rate of glucose transfer across the placenta, likely due to the altered maternal-fetal concentration gradient. There is also an overall increase in placental size in diabetic pregnancies, and babies who are large for gestational age are at increased risk for CHD.

In spite of the fact that some of the morphological changes in the placenta occur regardless of the level of maternal metabolic control, there is a correlation between the level of maternal hemoglobin A1c (HbA1c) levels and the risk of CHD (Gardiner et al., 2006). Increased HbA1c is correlated with a significant increase in myocardial shortening and flow velocities. This suggests that the etiology of diabetes-associated CHD is multifactorial, and while the placenta likely plays an important role, other factors including modifications of signal transduction pathways that influence cellular function play an important role. Indeed, maternal diabetes has been shown to result in inflammation and chronic stress (Radaelli et al., 2003). Hyperglycemia is also known to alter neural crest cell migration, which is a critical event in heart development (Reece, 1999). There may also be a hemodynamic influence on heart development, as fetal heart function in diabetic pregnancies may be altered as early as 12–14 weeks of development (Rizzo et al., 1995). CHD can be induced or made more severe by altered hemodynamic conditions (Midgett and Rugonyi, 2014; Midgett et al., 2017). Consequently, it is likely a complex interplay between many factors that increase the risk of CHD in the environment of maternal diabetes.

### Maternal Obesity

In keeping with the obesity epidemic, pre-pregnancy obesity has been on the rise, and an estimated 30% or greater of pregnant women were obese prior to becoming pregnant (Papachatzi et al., 2013). This has significant consequences to the health of the placenta, including an association with maternal hyperleptinemia and placental leptin resistance, and a decrease in the sodium-dependent neutral amino acid transporter, SNAT (Farley et al., 2010). Increased maternal leptin levels were associated with a down-regulation of leptin receptors in the syncytiotrophoblasts. This may be evidence of placental leptin resistance, and altered placental function, including reduced amino acid transport. In multiple studies, maternal obesity is also associated with placental lipotoxicity, oxidative stress, and inflammation (Challier et al., 2008; Roberts et al., 2009; Oliva et al., 2012). A study designed to identify the molecular basis of these dysfunctions that used RNA-sequencing on term placenta showed significant transcriptional differences between placentas from pre-pregnancy obese mothers compared to controls with pre-pregnancy normal body mass indices (BMI). Altered transcription was identified for genes related to lipid metabolism, hormone, and cytokine activity. There was a concomitant increase in placental lipids, a decrease in total antioxidant capacity, and a decrease in regulators of angiogenesis, and increases in markers of inflammation and oxidative stress (Saben et al., 2014).

In light of evidence that maternal obesity results in placental dysfunction, there is a likelihood that there may be an association with CHD in the offspring. However, at this point the association is circumstantial. Numerous epidemiological studies have revealed that pre-pregnancy maternal obesity is a risk factor for structural birth defects, including CHD (Watkins and Botto, 2001; Cedergren and Kallen, 2003; Gilboa et al., 2010), but none have yet shown a direct relationship to placental dysfunction. However, the epidemiological evidence of a relationship between maternal obesity and CHD is strong. In one of the largest studies to date, classification of mothers by BMI showed that overweight women (BMI 25–29) were not at an increased risk for having a fetus with CHD, whereas obese women (BMI ≥ 30) had a significantly increased risk (Mills et al., 2010). Risk further increased with the degree of obesity, where morbidly obese women (BMI ≥ 40) had a higher risk than those considered to be simply obese (BMI 30–39). The highest risk forms of CHD in obese women in both categories were varied and included atrial septal defects, hypoplastic left heart syndrome, aortic stenosis, pulmonary stenosis, and tetralogy of Fallot. Further studies are needed to link the occurrence of CHD with placental dysfunction, and to answer the vital question if pre-pregnancy weight loss will decrease risk of CHD.

## Hypertensive Disorders or Pregnancy

Preeclampsia is a specific form of placental insufficiency that threatens the health of both the fetus and the mother. Impaired vessel formation and/or function in the placenta limits placental perfusion and transfer of nutrients to the fetus as well as removal of waste products (Brennan et al., 2014). In the past few years there have been several registry and population-based studies seeking to determine if preeclampsia and other hypertensive disorders during pregnancy are associated with increased risk for CHD in the offspring. At this point in time three different studies have concluded that CHD is strongly associated with preeclampsia. A Norwegian registry-based study found an association between early-onset preeclampsia and clinically severe CHD (Brodwall et al., 2016). A separate, but concurrent study in Canada using hospital discharge records reported that early onset preeclampsia was associated with both clinically severe and clinically mild CHD, but the association was not as strong for late-onset preeclampsia (Auger et al., 2014). Most recently, a registry-based study from the Danish Hospital Discharge Register of nearly 2 million pregnancies found a similar association between all reported forms of CHD and preeclampsia (Boyd et al., 2017). In this study the pregnancies were subdivided by those with early preterm preeclampsia necessitating delivery early than 34 weeks, late preterm preeclampsia with delivery at 34–36 weeks, and term preeclampsia with delivery ≥37 weeks. All categories had an increased risk of CHD, with the greatest risk associated with early preterm preeclampsia. There was no association between maternal gestational hypertension and offspring CHD. Although the percentage risk assessments vary between studies, it is becoming clear that preeclampsia, particularly early term (<34 weeks) is associated with risk for CHD.

## Genetically Triggered CHD: Is there a Placental Contribution?

Although it is recognized that there is a heart–placenta axis, study of the functional overlap in genes expressed during the development of both organs is still incomplete. There are now numerous genetic mutations that have been shown to cause CHD in humans, particularly in familial CHD and/or syndromes that involve CHD as part of the phenotype (Gelb, 2013). In many of these the mutation is sufficient to cause the heart malformation as evidenced by mouse models where the mutation is introduced only into the cardiac lineage. However, what remains under-explored is if the severity of the heart defect is influenced by placental insufficiency where the mutation is not restricted to the heart. To date there are nearly 50 different genes that have been implicated as genetically associated with CHD in humans (Yuan et al., 2013). Most of these have been studied as conditional knockouts in the mouse, with altered gene expression restricted to cardiac precursors to show that the CHD is not secondary to other defects. However, most, if not all of the genes that have been shown to cause CHD in humans are also expressed in the placenta. The fetal microenvironment, which is largely controlled by the placenta, is worthy of consideration in the pathogenesis of CHD. This is particularly true when a genetic mutation is the direct underlying cause of CHD, but may also have an effect on the function of the placenta. For instance, mutations in *CITED2* cause cardiac septal defects and malrotation of the great arteries, with considerable phenotypic variability amongst individuals (Sperling et al., 2005). The mutations significantly reduce the ability of CITED2 to transrepress HIF1A and coactivate TFAP2C in the developing heart. Dysregulation of these early signaling pathways disrupt normal cardiovascular morphogenesis resulting in CHD. However, it is also known that CITED2 activity is required in trophoblasts for correct capillary patterning (Moreau et al., 2014). This appears to be mediated by PDGF signaling and results in abnormalities that are likely to affect nutrient transport to the fetus. What is not known is if the CHD-associated mutations affect CITED2 function in the placenta as well as the heart. The possibility that is mutation-induced defects in both the heart and placenta remains an intriguing possibility not only for *CITED2*, but for other CHD genes.

An important emerging theme in the genetic basis of CHD is the role of the primary cilia in heart development and association of cilia genes with CHD (Koefoed et al., 2014). Primary cilia are microtubule-based organelles present on the surface of almost all vertebrate animal cell types. The role of primary cilia is to respond to intracellular signals, particularly during development or tissue remodeling. During heart development, primary cilia are active in the SHF where they respond to sonic hedgehog signaling. Mutations in cilia component genes give rise to CHD (Burnicka-Turek et al., 2016), but that same structure is not present in extraembryonic tissues of the developing placenta (Bangs et al., 2015). Trophoblast and extraembryonic endoderm stem cells specifically lack primary cilia, which defines which cells in the placenta and yolk sac are incapable of responding to hedgehog signaling. A maternal cilia gene mutation it would be present in the cells of the maternally derived components of the decidua basalis. Epiblast-derived cells, such as the endothelial cells that line the fetal capillaries, could potentially be affected by the cilia gene mutation. Consequently, the overall impact on placental function could be significant. This means that CHD due to defects in cilia genes could have a substantial impact on placental insufficiency as well as the direct impact on the SHF. The relative contribution of the placenta on the nature and severity of the heart defect is worth further exploration.

A summary of the various placenta-based pathways to CHD is shown in **Table 1**.

**Table 1:** A summary of pathways, genes, and other factors that affect the fetal environment, the site of primary action, and the outcome of in terms of CHD.

Affected element	Primary site of action			Outcome
	Placenta	Placenta + Heart	Mother to placenta	
Wnt/β-catenin		√		CHD, multiple forms
Folate/MTHFR			√	Preeclampsia
Glucocorticoids		√		
IUGR	√			IUGR, Reduced cardiac function
MAP Kinase	√			CHD, multiple forms
PPARs	√			CHD, multiple forms
Hoxa13	√			Thin cardiac ventricular wall
SENP2	√			Hypoplastic heart chamber, thin cardiac ventricular wall, absent AV cushions
Hypoxia/VEGF		√		Multiple cardiac septal defects, outflow tract defects, right ventricle anomalies, thin ventricular wall
Maternal Diabetes			√	Outflow tract and cardiac septal defects
Maternal Obesity			?	Atrial septal defects, hypoplastic left heart, aortic stenosis, pulmonary stenosis, tetralogy of Fallot
Preeclampsia			√	CHD, multiple forms
CITED2		√		Cardiac septal defects, malrotation of the great arteries
Primary Cilia Genes		√		Cardiac septal defects

## Silent Congenital Heart Defects – Manifestation as Adult Disease

It is becoming increasingly recognized that not all CHD is recognizable at birth or even much later in life. Subtle defects that are not clinically evident at birth may remain silent for decades, but quietly increase susceptibility to cardiac disease with advancing age (Thornburg et al., 2010). This is also true in adults with recognized CHD where a cardiac defect has been repaired in infancy or early childhood, yet the person remains at increased risk for heart disease throughout life (Greutmann et al., 2015). However, the growing field of *Developmental Origins of Disease* further recognizes that clinically silent primary cardiac insufficiencies may result in an increased predisposition to cardiovascular disease in adult life. As discussed above, placental insufficiency can cause clinically significant CHD. However, this may just be the tip of the iceberg. In one study, cardiovascular assessment of children with IUGR showed structural and functional differences when compared to children with normal intrauterine growth (Crispi et al., 2010). Although some of these changes may occur postnatally, it is likely that the deficiencies had fetal origins. In particular, the IUGR children had some alterations of the shape of the heart with increased transversal diameters and more globular ventricles. They had a significantly reduced stroke volume, which was compensated for by increased heart rate to maintain output. They also had higher blood pressure and increased intima-medial thickness. The level of cardiovascular alterations correlated with the severity of IUGR. Although yet to be proven, this suggests that this silent CHD in IUGR children could congenitally predispose them to cardiovascular disease later in life. This correlates with numerous epidemiological studies showing that fetuses exposed to hypoxic or non-hypoxic placental insufficiency as suggested by low birth weight are more likely to have cardiovascular disease as adults (Thornburg et al., 2010).

## Conclusion

It has long been recognized that CHD is a complex trait with contributions from genetics, epigenetics, and environmental factors. It is clear from studies of families with Mendelian inheritance of CHD that it can be caused by single gene defects. It is equally clear from a combination of human and animal model studies that placental insufficiency alone can cause CHD. What is not so evident is how the interaction between genetic defects and placental insufficiency alters heart development and how that interaction contributes to CHD. Given the coordinated development of the placenta and the heart, and the placenta–heart genetic axis, there is considerable potential for contributions of primary defects from both the heart and placenta to have a major impact on the outcome of CHD. In particular, if there is a mutation in a gene that is involved in both heart and placenta development, there may be a synergistic effect that has its largest consequence on the heart.

Regardless of genetic underpinnings there are many other factors that can influence heart development and it is likely that for the most part CHD is a complex trait that has multifactorial origins. Maternal health is certainly a key factor that can significantly impact placental and fetal health. Common themes are emerging as to the underlying mechanisms of placental dysfunction, despite of the initiating cause. Inflammation and oxidative stress seem to be recurring downstream effects in placental dysfunction, often coupled with a multitude of other alterations such as changes in signal transduction profiles affecting vasculogenesis, cell migration, and a number of other key elements of development. However, the placenta remains central to all of these events and has a clear role in heart development and the pathogenesis of CHD.

The full range of CHD, including clinically silent defects, may be greater than previously recognized and much may be attributable to placental insufficiency. Even a small impact on placental function may have a synergistic effect on an already compromised fetus thereby increasing the severity of the heart defect, which remains an open issue in the field.

## Author Contributions

The author confirms being the sole contributor of this work and approved it for publication.

## Conflict of Interest Statement

The author declares that the research was conducted in the absence of any commercial or financial relationships that could be construed as a potential conflict of interest. The handling Editor declared a shared affiliation, though no other collaboration, with the author CM.
